# Expression of cyclooxygenase-2 (COX-2) in tumour and stroma compartments in cervical cancer: clinical implications

**DOI:** 10.1038/sj.bjc.6600578

**Published:** 2002-11-04

**Authors:** G Ferrandina, L Lauriola, G F Zannoni, M G Distefano, F Legge, V Salutari, M Gessi, N Maggiano, G Scambia, F O Ranelletti

**Affiliations:** Department of Obstetrics and Gynecology, Catholic University of the Sacred Heart, L.go F. Vito, 1, 00168 Rome, Italy; Department of Pathology, Catholic University of the Sacred Heart, L.go F. Vito, 1, 00168 Rome, Italy; Department of Histology, Catholic University of the Sacred Heart, L.goF. Vito, 1, 00168 Rome, Italy

**Keywords:** cervical cancer, COX-2, prognosis, chemotherapy response

## Abstract

This study aims at investigating the relationship between cyclooxygenase-2 expression in tumour *vs* stroma inflammatory compartment and its possible clinical role. The study included 99 stage IB-IV cervical cancer patients: immunostaining of tumour tissue sections was performed with rabbit antiserum against cyclooxygenase-2. CD3, CD4, CD8, CD25, Mast Cell Tryptase monoclonal antibodies were used to characterise stroma inflammatory cells in nine cervical tumours. An inverse relation was found between cyclooxygenase-2 levels (cyclooxygenase-2 IDV) of tumour *vs* stroma compartment (*r*=−0.44, *P*<0.0001). The percentage of cases showing high tumour/stromal cyclooxygenase-2 IDV ratio was significantly higher in patients who did not respond to treatment (93.3%) with respect to patients with partial (60.5%), and complete (43.7%) response (*P*= 0.009). Cases with a high tumour/stroma cyclooxygenase-2 IDV ratio had a shorter overall survival rate than cases with a low tumour/stroma cyclooxygenase-2 IDV (*P*<0.0001). In the multivariate analysis advanced stage and the status of tumour/stroma cyclooxygenase-2 IDV ratio retained an independent negative prognostic role. The proportion of CD3^+^, CD4^+^, and CD25^+^ cells was significantly lower in tumours with high tumour/stroma cyclooxygenase-2 IDV ratio, while a higher percentage of mast cells was detected in tumours showing high tumour/stroma cyclooxygenase-2 IDV ratio. Our study showed the usefulness of assessing cyclooxygenase-2 status both in tumour and stroma compartment in order to identify cervical cancer patients endowed with a very poor chance of response to neoadjuvant therapy and unfavourable prognosis.

*British Journal of Cancer* (2002) **87**, 1145–1152. doi:10.1038/sj.bjc.6600578 www.bjcancer.com

© 2002 Cancer Research UK

## 

Cyclooxygenase (COX), the key enzyme in the conversion of arachidonic acid to prostaglandins, exists in two isoforms namely COX-1, constitutively expressed in most tissues, and COX-2, which is inducible by growth factors, prostaglandins, and tumour promoters and associated with the inflammatory response (Williams and Dubois, 1996). It has been reported that COX-2 overexpression is associated with inhibition of apoptosis and host immune responses, and increased metastatic potential and neoangiogenesis (Tsujii and Dubois, 1995; Tsujii *et al*, 1998; Stolina *et al*, 2000). In this context, it is not surprising that COX-2 has been associated with parameters of tumour aggressiveness and unfavourable clinical outcome in several solid tumours (Ferrandina *et al*, 2002a; Tomozawa *et al*, 2000; Ristimaki *et al*, 2002). As far as cervical cancer is concerned, recent reports showed that high COX-2 expression is associated with diminished survival in cervical cancer patients administered radiotherapy (Gaffney *et al*, 2001). We also recently demonstrated that high COX-2 expression in tumour cells can identify cervical cancer patients with a poor chance of response to neoadjuvant chemotherapy and worse prognosis (Ferrandina *et al*, 2002b).

There is some evidence that, besides the expression in tumour cells, COX-2 is also expressed in stromal cellular elements such as endothelial cells, macrophages, and fibroblasts (Masferrer *et al*, 2000), stimulated T-lymphocytes and activated mast cells (Iniguez *et al*, 1999; Hundley *et al*, 2001).

The potential role of COX-2 expression in stroma cells has been first suggested by Williams *et al* (2000), who showed a dramatic inhibition of tumour growth and angiogenesis in lung carcinoma cells grafted into homozygous COX-2^−/−^ mice.

Based on our preliminary observations about the presence of COX-2 staining in the stroma compartment of cervical tumours we were then prompted at providing a more in depth analysis of (i) COX-2 content in the stroma inflammatory cellular elements of this neoplasia, (ii) the relationship between COX-2 expression in tumour cells *vs* stroma inflammatory compartment, and (iii) the possible clinical role of COX-2 expression according to the cellular compartment of staining.

In addition, an immunophenotypic characterization of stroma inflammatory cells was carried out in a series of cervical tumours.

## PATIENTS AND METHODS

The study included 99 stage IB-IV cervical cancer patients consecutively admitted to the Department of Obstetrics and Gynecology, Division of Gynecologic Oncology, Catholic University of Rome between November 1995 and September 2001. Median age was 51 years (range 24–76). The clinico-pathological characteristics are summarised in Table 1

**Table 1:**
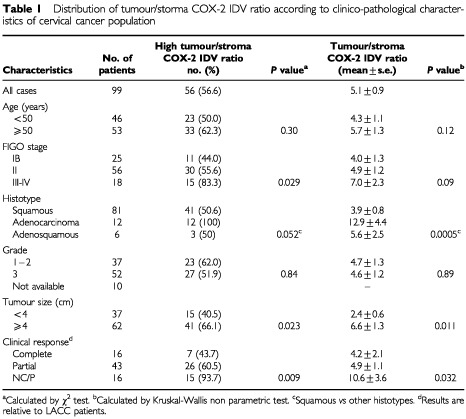
Distribution of tumour/storma COX-2 IDV ratio according to clinico-pathological characteristics of cervical cancer population

. The clinical management of our patient population was as previously described (Ferrandina *et al*, 2002b): cases with early stage disease (FIGO Stage IB-IIA, major tumour diameter less than 4 cm) were primarily submitted to radical surgery (*n*=24), whereas locally advanced cancer cases (*n*=75) were first administered neoadjuvant cisplatin-based treatment (cisplatin dose 100 mg m^2^ every 3 weeks for 2–3 courses). In case of clinical response, assessed by the above described procedures and recorded according to WHO response evaluation criteria (World Health Organization, 1979), LACC patients were submitted to radical surgery (*n*=61): operative technique consisted of type II–IV radical hysterectomy, and systematic pelvic lymphadenectomy. An anterior exenteration was performed in two cases with persisting involvement of the bladder after neoadjuvant treatment. Para-aortic lymphadenectomy up to the level of inferior mesenteric artery was performed in high risk patients as previously reported (Benedetti-Panici *et al*, 1996). Patients showing clinical no change/progression during neoadjuvant treatment were subjected to exclusive radiotherapy.

### Antibodies

The following monoclonal and policlonal antibodies were used: anti-CD3 (clone PS1; 1 : 100), anti-CD4 (clone 1F6; 1 : 50), anti-CD8 (clone 4B11; 1 : 50), anti-CD25 (clone 4C9; 1 : 100), all from Ylem (Avezzano, Italy); anti-Mast Cell Tryptase (clone AA1, 1 : 100 (DAKO, Glostrup, Denmark); anti-COX 2 rabbit policlonal antibody (1 : 300 Cayman, Ann Arbor, MI, USA).

### Immunohistochemical studies

Tumour tissues biopsies were performed under colposcopic examination. Immunohistochemistry of COX-2 was performed as previously described (Ferrandina *et al*, 2002b). In the studies of phenotyping of stromal inflammatory cells, serial sections of representative blocks were cut from patients with either high (>1) or low (⩽1) COX-2 positive tumour/stroma ratio (see below). In order to quantitate the percentage of COX-2 stained cell subpopulations with respect to inflammatory cells of the stroma, we used the immunoperoxidase technique performed on consecutive sections, since it allows by nuclear counterstaining with hematoxylin to better recognise stromal cells also by their morphological characteristics.

Negative controls were performed using non immunised rabbit serum and/or omitting the primary antibodies. As positive controls for COX-2 antibody, COX-2 positive Hep-2 squamous cancer cells and COX-2 positive squamous cancer tissue specimens (Ranelletti *et al*, 2001) were always run in the assay. As positive controls for the other antibodies both lymph-nodes and thymus were used.

### Double-labelling subtraction immunostaining

Double-labelling subtraction immunostaining was carried out as previously reported (Yamada *et al*, 1998). Briefly, after routine immunostaining for COX-2, the slide was treated with 3-amino-9 ethylcarbazole (Sigma, St. Louis, MO, USA). The slide was rapidly mounted using an aqueous mounting medium and digital images of microscopic fields acquired by Nikon Coolpix 950 digital camera (Nikon Instruments, Florence, Italy) after registering the X-Y coordinate of the acquired field in the slide. Then the slide was immersed in Ca^2+^-Mg^2+^-free PBS (pH 7.2), and washed with PBS. The slide was immersed for 10 s in 99.8% (vol/vol) methyl alcohol and then exhaustively washed with PBS. The section was treated in microwave owen using the Dako ChemMate detection kit (DAKO) and then incubated with anti-mast cell tryptase antibody. The immunoreaction was developed by fast blue and the slide was remounted. The exact area on the same slide was acquired by the digital camera and compared with the first image. As a control, after the first immunostaining with anti-COX-2 antibody, the slide was processed, as reported above, and then treated with the streptavidin-biotin-alkaline phosphatase complex kit, omitting the anti-mast cell tryptase antibody.

### Quantification of immunohistochemical staining

The intensity of immunohistochemical staining was evaluated as previously reported (Ranelletti *et al*, 2001) by the image analysis based on Photoshop (Adobe System, San Jose, CA, USA) together with ‘The image processing toolkit’ (CRC Press, Boca Raton, FL, USA). The integrated density values (IDV) of the immunostaining was calculated as the product of the mean density value of the immunoreactive regions by the percentage of the immunostained tumour or stroma components. Inflammatory cell count in the tumour stroma was performed by chosing five corresponding ×20 fields, from each of six serial tissue sections (one for each antibody), so as to best reflect the overall immunostaining of the tumour stroma contained in the entire slide. The files, acquired with a Nikon Coolpix 950 digital camera, were opened in Photoshop using a Macintosh G3 workstation (Apple, Cupertino, CA, USA). Both immunostained and negative cells within a superimposed grid of 0.022 mm^2^ were counted. Two cell counts for each digital image were done by moving the grid over representative stromal areas. The total cell number/stromal area was calculated by averaging cell counts from each section and from the six consecutive sections (*n*=60). The number of immunostained cells, relative to each phenotype, was calculated by averaging cell counts from two grid areas from five ×20 fields (*n*=10). The results were reported as mean±s.e.

### Statistical analysis

The χ^2^-test was used to analyse the distribution of COX-2 positive cases according to several clinico-pathological features. Kruskal–Wallis non parametric test was used to analyse the distribution of tumour/stroma COX-2 integrated density values according to clinico-pathological variables. Medians and life tables were computed using the product-limit estimate by the Kaplan and Meier method (Kaplan and Meyer, 1958) and the log-rank test was employed to assess the statistical significance (Mantel, 1966). The prognostic role of COX-2 as a continuous variable, was also analysed by means of the Cox proportional hazard model (Cox, 1972). A Cox's regression model with stepwise variable selection (Cox, 1972) and multiple logistic analysis (Cox, 1970) were used to analyse the role of clinico-pathological parameters and COX-2 staining as prognostic factors and predictors of response to neo-adjuvant treatment. Wald statistics for coefficient comparison and the Joint significance test were also performed in order to compare the coefficients of relative risk of death for tumour COX-2 positivity *vs* tumour/stroma COX-2 IDV ratio positivity and to evaluate the weight of the status of tumour COX-2 and tumour/stroma COX-2 IDV ratio in the survival regression model after excluding each of them.

Statistical analysis was carried out using SOLO (BMDP Statistical Software, Los Angeles, CA, USA) and Statview survival tools (Abacus Concepts- Inc- Berkeley CA, USA).

## RESULTS

### Cox-2 immunostaining

Figure 1A

**Figure 1:**
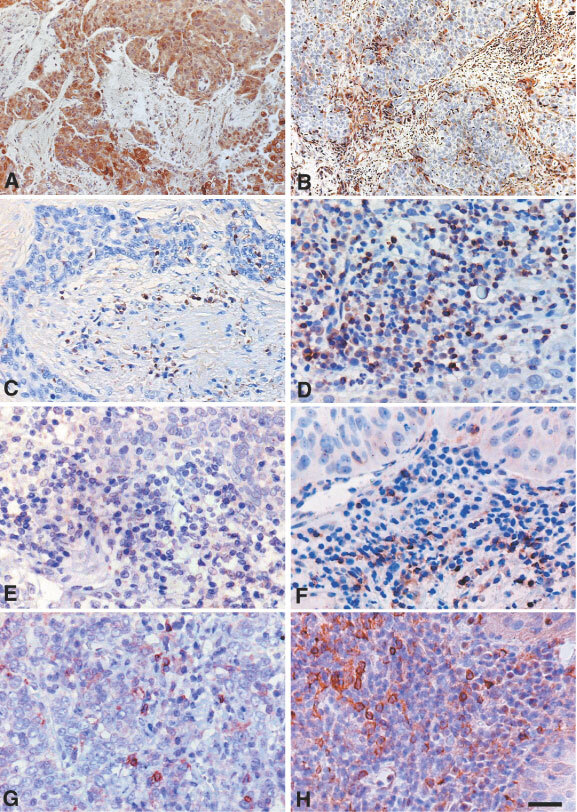
(**A**) Squamous cervical cancer with intense COX-2 immunoreaction in both cytoplasm and nuclei of tumour cells. Scattered cells in the stromal compartment are stained. (**B**) COX-2 negative tumour showing intense COX-2 staining in the stroma inflammatory compartment. CD3, CD4, CD25, and tryptase immunoreaction in tumours showing high (**C**, **E**, **G**) *vs* low (**D**, **F**, **H**) tumour/stroma COX-2 IDV ratio. Bar=50 μm (**A**,**B**); Bar=25 μm (**C**–**H**).

and B shows COX-2 immunoreaction in two primary squamous cervical tumours. COX-2 immunostaining was observed both in the tumour cells as well as in the stroma inflammatory compartment of the tumour. Interestingly, in the presence of strong COX-2 staining in tumour cells, only barely detectable COX-2 immunoreaction was observed in the stroma inflammatory compartment (Figure 1A). On the other hand, a large amount of stroma inflammatory component showing positive COX-2 immunostaining was frequently detected in association with low or absent COX-2 staining in tumour cells (Figure 1B).

In the whole series, COX-2 integrated density values in the tumour component ranged from 1.2 to 82.3 with mean±s.e. values 25.5±2.2. COX-2 integrated density values in the stromal component range from 0.9 to 96.0 with mean+s.e. values of 20.0±1.9.

A statistically significant inverse relation was found between COX-2 IDV of tumour *vs* COX-2 IDV in the stroma compartment (*r*=−0.44, *P*<0.0001) (data not shown).

For this reason, the ratio between COX-2 IDV in the tumour *vs* COX-2 IDV in the stroma component was used in order to normalise the COX-2 expression in each case, and to categorise tumours according to low *vs* high COX-2 content. The tumour/stroma COX-2 IDV ratio range from 0.03 to 48.2 (mean±s.e.=5.1±0.9). The ratio of ⩽1 was used to indicate cervical tumours with COX-2 expression in the tumour component lower or equivalent to COX-2 expression in the stroma.

According to the chosen cut off value, 56 out of 99 (56.6%) were scored as having a high (>1) tumour/stroma COX-2 IDV ratio.

### Correlation with clinico-pathological parameters

High COX-2 IDV in the tumour compartment were shown to be significantly associated with larger volume of the tumour and more aggressive histotype while COX-2 IDV in the tumour stroma showed the opposite pattern (data not shown).

The percentage of cases with high tumour/stroma COX-2 IDV ratio increased from 44.0% in stage I, through 55.6% in stage II, to 83.3% in stage III–IV cases (*P* value=0.029). Moreover, cases with high tumour/stroma COX-2 IDV ratio were more frequently observed in cases with tumour volume ⩾4 cm than in smaller tumours (66.1% *vs* 40.5%) (*P* value=0.023). No association with age, and grade of differentiation was found (Table 1).

Similarly, higher tumour/stroma COX-2 IDV ratio was found in stage III-IV with respect to stage I–II cases (*P* value=0.09), in adenocarcinoma and adenosquamous carcinoma versus squamous cell (*P* value=0.0005), in tumours ⩾4 cm *vs* smaller tumours (*P* value=0.011).

Metastatic lymph node involvement was found in 14 out of 69 (20.3%) cases: the percentage of COX-2 tumour positivity was 28.6% in lymph node positive with respect to 35.7% in lymph node negative cases (difference not significant).

### COX-2 status and response to neoadjuvant treatment

The percentage of cases showing tumour COX-2 positivity was significantly higher in patients who did not respond to treatment (87.5%) with respect to patients with partial (46.5%), and complete (31.2%) response (*P*=0.003). Similar results were found considering COX-2 IDV ratio as covariate, according to clinical response (*P*=0.007) (data not shown). On the other hand, the percentage of cases showing COX-2 IDV positivity in the stroma was not correlated *per se* with response to treatment (data not shown).

The percentage of cases showing high tumour/stromal COX-2 IDV ratio was significantly higher in patients who did not respond to treatment (93.3%) with respect to patients with partial (60.5%), and complete (43.7%) response (*P*=0.009). Similar results were found considering mean COX-2 IDV ratio according to clinical response (Table 1).

In the univariate analysis advanced FIGO stage, and high tumour/stroma COX-2 IDV ratio proved to associated with poor chance of response to neoadjuvant therapy (complete/partial *vs* no response). When logistic regression was applied, FIGO stage (χ^2^=7.0, *P*=0.008) and tumour/stroma COX-2 IDV ratio (χ^2^=4.1, *P*=0.0042) retained an independent role in predicting a poor chance of response to treatment.

### Survival analysis

Follow up data were available for 99 patients. As of December 2001, the median follow up was 23 months (range=3–73). During the follow up period, 21 out of 99 (21.2%) patients died of disease. For analysis of survival COX-2 status in tumour cells was defined according to the cut-off used in our previous study (Ferrandina *et al*, 2002b) and corresponding to the mean of COX-IDV. Similarly, COX-2 status in stroma inflammatory cells was defined according to the cut-off of 20.0 corresponding to mean COX-2 IDV.

**Figure 2:**
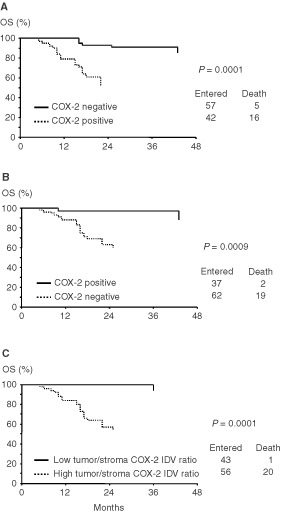
Overall survival rate according to the status of COX-2 in tumour cells (**A**), in stroma inflammatory cells (**B**), and according to the ratio between tumour/stroma COX-2 IDV (**C**) in 99 cervical cancer patients.

Cases showing COX-2 positive reaction in tumour cells (Figure 2A

) showed a shorter OS than COX-2 negative cases: in particular, the 3-year OS was 53% (confidence intervals 95% – CI 95% – 35–71) with respect to COX-2 negative cases who had a 3-year OS of 87% (CI 95%=75–99) (*P*=0.0001). COX-2 IDV in tumour cells were directly associated with risk of death, using COX-2 values as a continuous covariate (χ^2^=16.6, *P*=0.0001) (data not shown).

On the other side, cases showing COX-2 positive reaction in stroma inflammatory cells showed a longer OS than COX-2 negative cases: in particular, the 3-year OS was 91% (CI 95%=79–101) in COX-2 positive with respect to COX-2 negative cases who had a 3-year OS of 59% (CI 95%=46–72) (*P*=0.010) (Figure 2B). COX-2 IDV in stroma inflammatory cells were inversely associated with risk of death, as assessed by COX analysis using COX-2 values as a continuous covariate (χ^2^=8.2, *P*=0.004) (data not shown).

**Figure 3:**
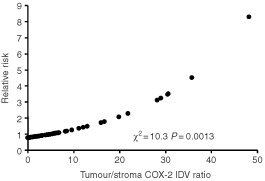
Plot of the estimate of the relative risk of death as a prediction of COX-2 integrated density values, calculated by COX's hazard regression model in cervical cancer patients.

Cases with a high tumour/stroma COX-2 IDV ratio had a shorter OS than cases with a low tumour/stroma COX-2 IDV ratio: in particular, all deaths of disease but one occurred in the former group (*P*<0.0001) (Figure 2C). Similar results were observed in the subgroup of LACC patients (data not shown). The use of an arbitrary cut-off to distinguish cases with high *vs* low tumour/stroma COX-2 IDV ratio is unlikely to have introduced any bias, since a direct association between tumour/stroma COX-2 IDV ratio values and risk of death in the overall series was found (χ^2^=10.3; *P*=0.0013) using the ratio between tumour/stromal COX-2 IDV as continuous covariate (Figure 3

).

In multivariate analysis the positivity of tumour COX-2 retained an independent negative prognostic role for OS (*P*=0.0004), while the positivity of stromal COX-2 showed a trend toward a favourable role (*P*=0.056). Similar results were obtained when using tumour COX-2 and stromal COX-2 as continuous variables (data not shown).

As shown in Table 2

**Table 2:**
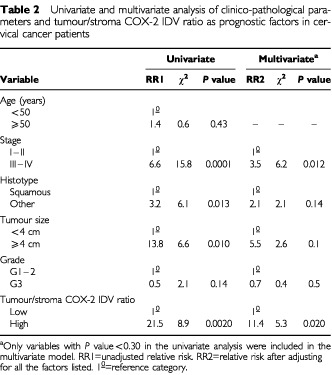
Univariate and multivariate analysis of clinico-pathological parameters and tumour/stroma COX-2 IDV ratio as prognostic factors in cervical cancer patients

the status of tumour/stroma COX-2 IDV ratio together with advanced stage of disease, retained an independent negative prognostic role for OS (Table 2). Similar results were obtained in multivariate analysis considering the values of tumour/stroma COX-2 IDV ratio as continuous variable in the whole series as well as in LACC patients (data not shown). Finally, in order to evaluate the weight of the status of tumour COX-2 and tumour/stroma COX-2 IDV ratio in the survival regression model, we tested the significance of excluding either the co-variate tumour COX-2 positivity or tumour/stroma COX-2 IDV positivity from the model. Indeed, only the tumour/stroma COX-2 IDV gave a statistically significant contribution to the model (likelihood ratio: χ^2^ =7.6, *P*=0.0058)

Moreover, the comparison between the coefficients of relative risk of death for tumour COX-2 positivity *vs* tumour/stroma COX-2 IDV positivity was also analysed in order to test whether a statistically significant difference exists beween the two coefficients. Indeed, in the Cox's proportional hazard model we found that the relative risk of death in patients having a tumour/stroma COX-2 IDV ratio >1 positivity showed a trend to be higher (relative risk 18.72, 95% CI=1.94–181.0) than that of patients with tumour COX-2 (relative risk 1.12, 95% CI=0.33–84.0) (Wald statistics: χ^2^=3.292; d.f.=1, *P*=0.069).

### Immunophenotypic characterisation

**Figure 4:**
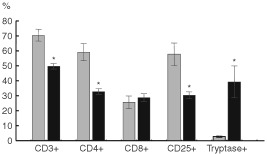
Percentage of CD3^+^, CD4^+^, CD8^+^, CD25^+^, and tryptase positive cells in stroma inflammatory infiltrate of cervical tumours from cases with low (gray columns) *vs* high (black columns) tumour/stroma COX-2 IDV ratio (results are expressed as mean±s.e.; *significant at *P*<0.05).

Quantification of the total amount of cells as well as the expression of CD3, CD4, CD8, CD25 and tryptase in the stromal inflammatory compartment were analysed in nine squamous cervical cancers randomly chosen among cases with high (>1) *vs* low (⩽1) tumour/stroma COX-2 IDV ratio. The total amount of cells observed in the stromal inflammatory compartment per unit area was significantly lower in cervical cancer showing high *vs* low tumour/stroma COX-2 IDV ratio (mean±s.e.=54.40±6.49 *vs* 121.67±13.30, *P*=0.014). Moreover, analysis of the data of CD3, CD4, CD25, and tryptase immunostaining, represented in Figure 1C–H and summarised in Figure 4

, shows that the proportion of CD3^+^, CD4^+^, and CD25^+^ cells is significantly lower in tumours showing COX-2 positive *vs* COX-2 negative tumour cells (*P*=0.014; 0.025, and 0.014, for CD3, CD4, and CD25, respectively). On the contrary, a higher percentage of tryptase positive mast cells was detected in stromal inflammatory cells from tumours with COX-2 positive *vs* COX-2 negative tumour cells (*P*=0.013). Interestingly, mast cells endowed with positive tryptase immunostaining were more frequently observed in tumours showing high versus low tumour/stroma COX-2 IDV ratio (*P*=0.014).

**Figure 5:**
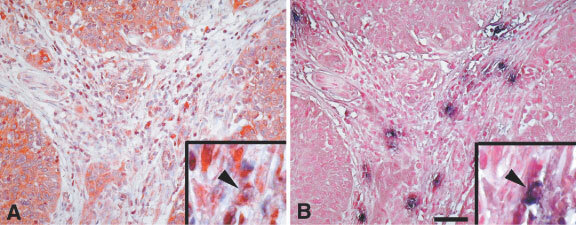
Tryptase staining in mast cells. Double labelling subtraction consecutive immunostaining of the same COX-2 positive tumour section by anti-COX-2 and anti-tryptase antibodies showed that the vast majority (>70%) of tryptase positive mast cells also express COX-2, insert). Bar: 50 μm.

Double labelling subtraction consecutive immunostaining of the same COX-2 positive tumour section by anti-COX-2 and anti-tryptase antibodies showed that the vast majority (>70%) of tryptase positive mast cells also express COX-2 (Figure 5

, insert).

## DISCUSSION

In this study, we first reported that in cervical tumours the expression of COX-2 in the stroma inflammatory cells and its relationship with COX-2 expression in tumour cells can be clinically relevant. An inverse relationship between COX-2 expression in tumour cells and the amount of the stroma inflammatory infiltrate in the tumour has been detected.

Moreover, COX-2 positive tumours showed a lower percentage of COX-2 positive stromal cells/unit area. This finding seems to be a distinctive morphologic feature of cervical tumours since we could not find such histopathological pattern in ovarian (Ferrandina *et al*, 2002a) and endometrial cancer (manuscript submitted), and suggests that tissue specific factors may intervene to regulate tumour/host interactions and characterise the amount and possibly the composition of stromal cellular infiltrate. The association between the presence of an abundant lymphocytic infiltrate in the stroma and better clinical outcome has been already highlighted in cervical cancer (Chao *et al*, 1999) as well as other solid tumours (Zeid and Muller, 1993) and associated with the potentiation of antitumour immune response, although the subpopulations of immune cells involved have been only partially clarified (Chao *et al*, 1999; Santin *et al*, 2001). We showed a lower percentage of CD3^+^, CD4^+^ and CD25^+^ T cells in tumours with COX-2 positive immunostaining in tumour cells suggesting that the expression of high levels of COX-2 in tumour cells can play a major role in inhibiting host immune functions. In this context, it is noteworthy that COX-2 can up-regulate the immunosuppressive cytokine IL10, while reducing the production of IL12, which is critical for cell mediated anti-tumour immunity (Stolina *et al*, 2000).

We also found a higher number of tryptase positive mast cells in tumours showing intense *vs* low COX-2 staining in tumour cells. Moreover, a large proportion of stromal mast cells was found to express COX-2, suggesting that they are in an activated state, as supported by the observations that induction of COX-2 at mRNA and protein level occurs in antigen-activated mast cells (Hundley *et al*, 2001).

Tryptase positive mast cells have been reported to increase from normal cervical tissue through cervical dysplasias to invasive carcinoma (Bribiesca-Benitez *et al*, 2001) and suggested to be involved in the promotion of tumour angiogenesis through the secretion of tryptase, a serin-protease able to exert a potent pro-angiogenic activity in human malignancies (Kankkunen *et al*, 1997) .

Therefore, the presence of high COX-2 expression in cervical tumour cells seems to be associated with a scarce cellular infiltrate in the stroma and, notably, with a lower proportion of immunoregulatory cells and a high proportion of tryptase positive mast cells. At present, our data do not allow to indicate the mechanisms and direction of tumour/host interaction relative to COX-2 expression. The possible active role of COX-2 expression in the stromal compartment of the tumour in terms of tumour cell/ stromal cell cross-talk has been recently highlighted (Prescott, 2000). In particular, Lehay *et al* (2002) showed that induction of apoptosis by COX-2 inhibitors in angiogenic cells is associated with antitumour activity, suggesting that COX-2 expression in stromal elements such as the vasculature could play a major role in tumour biology. This issue is relevant in order to understand the role of COX-2 according to different compartments, and warrants further investigations also by a thorough characterisation of the various cellular subtypes (lymphocytes, endothelial cells, macrophages, etc) in tumour stroma.

From a clinical point of view, it is noteworthy that, while COX-2 in tumour cells identifies cervical cancer patients with unfavourable prognosis, COX-2 expression in stroma inflammatory compartment is associated with better clinical outcome, suggesting that COX-2 positive stromal cells can play a role in reducing tumour cell aggressiveness. Therefore, the balance between COX-2 expression in the tumour and in the stroma area, rather than the independent evaluation of each compartment, could offer additional prognostic information. In this context, the use of the ratio between COX-2 in the tumour cells and COX-2 in the stroma cells, seems to be a more effective estimate of the cross-talk occurring between tumour tissue and its stromal compartment (Prescott, 2000; Williams *et al*, 2000). This ratio represents a valuable tool to normalise the amount of COX-2 expression in the tumour on the basis of the status of its stromal component, and to minimize the bias inherent to the use of an arbitrary cut-off.

Indeed, we showed that the ratio between COX-2 in the tumour cells and COX-2 in the stroma cells was very effective in distinguishing patients with low *vs* high risk of death of disease both in univariate and multivariate analysis. Finally, a very strong correlation between both tumour COX-2 expression and tumour/stroma COX-2 IDV ratio were shown to be highly correlated with response to chemotherapy while, although high COX-2 expression in the stroma was significantly associated with better survival, it failed to directly correlate with response to treatment.

In conclusion, our study showed the usefulness of assessing COX-2 status both in tumour and stroma compartment in order to identify cervical cancer patients endowed with a very poor chance of response to neoadjuvant therapy and unfavourable prognosis. This group of patients could possibly benefit of more individualised treatments, such as selective COX-2 inhibitors, which are already approved for treatment of familial colorectal adenomatous polyposis, and started to be explored in Phase I–II studies (Snyderman, 2001).
